# Sensing of transposable elements by the antiviral innate immune system

**DOI:** 10.1261/rna.078721.121

**Published:** 2021-07

**Authors:** Ana Gázquez-Gutiérrez, Jeroen Witteveldt, Sara R. Heras, Sara Macias

**Affiliations:** 1 GENYO Centre for Genomics and Oncological Research, Pfizer University of Granada, Andalusian Regional Government, PTS Granada, 18016, Granada, Spain; 2 Department of Biochemistry and Molecular Biology II, Faculty of Pharmacy, University of Granada, Campus Universitario de Cartuja, 18071, Granada, Spain; 3 Institute of Immunology and Infection Research, School of Biological Sciences, University of Edinburgh, EH9 3FL Edinburgh, United Kingdom

**Keywords:** mobile genetic elements, transposable elements, type I interferon, nucleic acid sensing, antiviral immunity

## Abstract

Around half of the genomes in mammals are composed of transposable elements (TEs) such as DNA transposons and retrotransposons. Several mechanisms have evolved to prevent their activity and the detrimental impact of their insertional mutagenesis. Despite these potentially negative effects, TEs are essential drivers of evolution, and in certain settings, beneficial to their hosts. For instance, TEs have rewired the antiviral gene regulatory network and are required for early embryonic development. However, due to structural similarities between TE-derived and viral nucleic acids, cells can misidentify TEs as invading viruses and trigger the major antiviral innate immune pathway, the type I interferon (IFN) response. This review will focus on the different settings in which the role of TE-mediated IFN activation has been documented, including cancer and senescence. Importantly, TEs may also play a causative role in the development of complex autoimmune diseases characterized by constitutive type I IFN activation. All these observations suggest the presence of strong but opposing forces driving the coevolution of TEs and antiviral defense. A better biological understanding of the TE replicative cycle as well as of the antiviral nucleic acid sensing mechanisms will provide insights into how these two biological processes interact and will help to design better strategies to treat human diseases characterized by aberrant TE expression and/or type I IFN activation.

## INTRODUCTION

Transposable elements (TEs) are a driving force in evolution but pose a potential threat to the fitness of the host by disrupting the function and/or expression of genes surrounding novel insertions or by promoting chromosomal rearrangements (Garcia-Perez et al. 2016; Bourque et al. 2018). To prevent these issues, TEs expression is generally repressed, either at the epigenetic, transcriptional, or post-transcriptional level. Intriguingly, in specific cellular contexts and developmental stages TE expression is activated and may have beneficial consequences. For instance, the RNA derived from the major mammalian retrotransposon, LINE-1, appears to be essential for maintaining the pluripotency-specific gene expression programs (Percharde et al. 2018). During viral infections the expression of TEs is also up-regulated, but it remains unclear if this is required to establish a proficient antiviral state, or is a secondary effect of the robust transcriptional response triggered upon infections (Macchietto et al. 2020; Srinivasachar Badarinarayan et al. 2020). This review will focus on the role of mammalian TE-derived nucleic acids in stimulating the antiviral innate immune response. We provide a brief overview of the major mammalian TEs and innate antiviral defence mechanisms to understand the evolutionary interactions between TEs and antiviral defence.

## MOUSE AND HUMAN TRANSPOSABLE ELEMENTS: AN OVERVIEW

**FIGURE 1.**
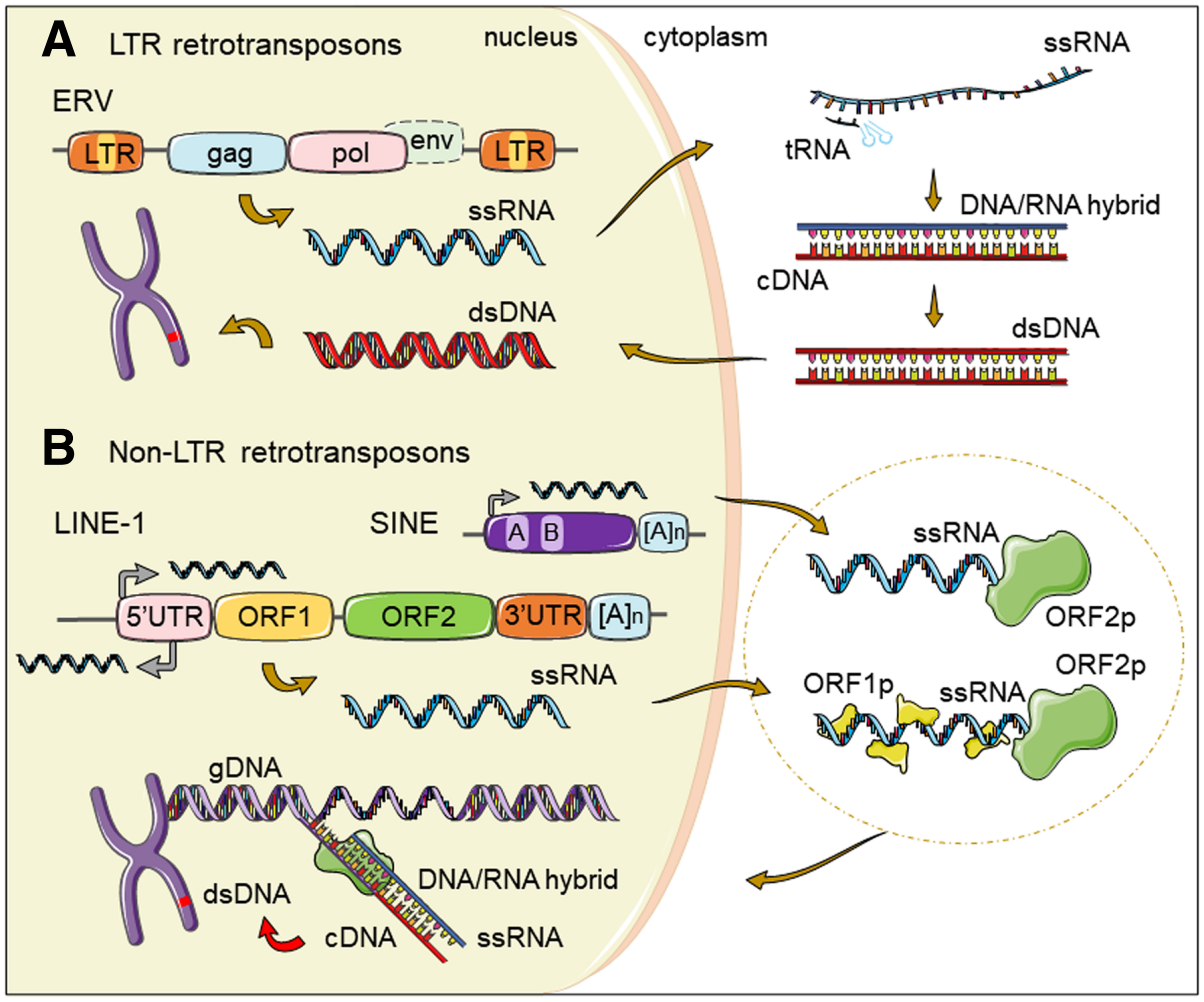
Mammalian LTR versus non-LTR retrotransposon replicative cycles. (*A*) ERVs are flanked by long-terminal repeats (LTRs) and encode Gag and Pol proteins while lacking a functional *env* gene. After transcription, the single-stranded RNA (ssRNA) from ERVs is exported to the cytoplasm. A transfer RNA (tRNA) binds the ssRNA molecule and serves as a primer for discontinuous reverse transcription to generate a dsDNA copy. The dsDNA is then imported to the nucleus where it integrates in the genome. (*B*) SINE elements, which include human Alu and mouse B1/B2 elements, typically contain an RNA pol III transcription start site (black arrow), sequences required for transcription (A and B boxes) and a terminal poly(A) repeat [(A)n]. *Below* SINEs, the structure of a full-length LINE-1 element is represented, containing a 5′ untranslated region (5′UTR), with sense and antisense RNA pol II promoter transcription start sites (black arrows), two nonoverlapping open reading frames (ORF1 and ORF2) and a short 3′UTR with a poly(A) tail. The ssRNA from LINEs and SINEs is exported to the cytoplasm and bound by LINE-1 encoded proteins, forming a ribonucleoparticle that is imported into the nucleus. ORF2p endonuclease domain nicks the genomic DNA (gDNA) at a target site, and the exposed 3′ OH group is used by the reverse transcriptase domain to prime the reverse transcription of the RNA template. This mechanism, called target-primed reverse transcription (TPRT), generates an RNA/DNA hybrid intermediate. In the next steps, which are still not completely understood, a new dsDNA copy of the SINE or LINE element is formed and integrated into a new genomic location.

TEs can be defined as DNA fragments that are able to mobilize from one place to another within the genome and are divided in two major groups based on their mobilization mechanism (Finnegan 1989; Beck et al. 2011). On the one hand, DNA transposons (or class II elements) mobilize through a “cut and paste” mechanism, in which the transposon is excised from the donor site and inserted in a different genomic location. They comprise ∼3% of the human genome and are no longer competent for mobilization (Lander et al. 2001). On the other hand, retrotransposons (or class I elements) mobilize through a “copy and paste” mechanism that involves the reverse transcription of an RNA intermediate to generate a new copy of the element. Retrotransposons can be further divided based on their structure and the mechanism of reverse transcription. In the case of retrotransposons with long terminal repeats, LTR retrotransposons including endogenous retroviruses (ERVs), the double-stranded DNA (dsDNA) copy is generated in the cytoplasm using a tRNA as a primer for reverse transcription. Once synthesized, the dsDNA travels to the nucleus, where it is inserted into the genome by the integrase enzyme (Fig. 1A; Bannert and Kurth 2006). In contrast, non-LTR retrotransposons, which include long interspersed elements (LINEs) and short interspersed elements (SINEs), have developed a different strategy: A LINE-1-encoded endonuclease nicks the target DNA generating an exposed 3′-OH that serves as a primer to produce a new cDNA molecule at the site of integration. This mechanism is called target-primed reverse transcription (TPRT) (Fig. 1B; Luan et al. 1993; Cost et al. 2002).

In terms of genomic structure, ERVs resemble retroviruses. ERVs contain *gag* and *pol* genes which encode structural and enzymatic proteins, including the reverse transcriptase, but lack functional envelope *env* genes. Although human ERVs (HERVs) comprise 8% of our genome, to date, no competent HERVs have been described. However, these elements impact gene expression in many ways, acting as enhancers or via the production of long noncoding RNAs (lncRNAs) regulating pluripotency maintenance (Garcia-Perez et al. 2016). In mice, ERVs account for 10% of the genome and, in contrast to humans, several subfamilies are actively mobilized, including IAP (intracisternal A-particle) and MusD (Mus-type D related retroviruses) elements, among others (Lueders and Kuff 1977).

Around 40% of the human and mouse genomes are made of non-LTR retrotransposons and in both species, several families are active. LINEs are autonomous (i.e., they encode the proteins required for their mobilization), while SINEs require the proteins encoded by LINE elements to mobilize (Moran et al. 1996; Dewannieux et al. 2003; Hancks et al. 2011; Richardson et al. 2015). Three distant families of LINEs, LINE-1, LINE-2, and LINE-3, account for 21% of the human genome (Lander et al. 2001); however, only elements belonging to the LINE-1 family have remained active (Brouha et al. 2003; Khan et al. 2006). A competent LINE-1 is typically ∼6 kb long and contains a 5′ untranslated region (5′UTR), two nonoverlapping open reading frames (ORF1 and ORF2) and a short 3′UTR containing a poly(A) tail. ORF1 encodes an RNA binding protein with nucleic acid chaperone activity, whereas ORF2 encodes a protein with endonuclease (EN) and reverse transcriptase (RT) activities. Both ORFs are required for LINE-1 retrotransposition, but only ORF2 is essential for mobilization (for review, see Richardson et al. 2015). The LINE-1 5′UTR has an internal RNA polymerase II promoter that directs transcription of the full-length element and an antisense promoter (Swergold 1990; Speek 2001). Antisense transcription can lead to chimeric transcripts containing a portion of the LINE-1 5′UTR and locus-specific upstream genomic sequences. Furthermore, the antisense promoter drives the expression of a small peptide, ORF0, whose function is still unclear (Denli et al. 2015). It is predicted that all 5′UTRs from the 16 different primate-specific amplifying LINE-1 subfamilies (L1PA16–L1PA1) have the ability to drive transcription, despite lacking sequence homology, while the antisense promoter region is only conserved among the youngest families (L1PA6–L1PA1) (Khan et al. 2006). However, the vast majority of LINE-1 copies in mammalian genomes are 5′ truncated and therefore inactive and only ∼80–100 copies from the youngest LINE-1 subfamily L1PA1, also called L1Hs, have retained the potential to mobilize (Brouha et al. 2003; Beck et al. 2011). In mice, around 3000 copies of mouse LINE-1, from subfamilies A, T_F_, and G_F,_ remain active. The 5′UTR of mouse LINEs consists of monomeric repeats and contains an RNA polymerase II sense strand promoter, while the antisense promoter is located at ORF1 (Deberardinis and Kazazian 1999; Li et al. 2014). The bidirectional transcription of opposed LINE-1 retrotransposon sequences could potentially result in the formation of double-stranded RNAs (dsRNAs), as previously suggested (Yang and Kazazian 2006). Although at the structural level mouse LINE-1s are similar to human LINEs, they differ at the 5′UTR sequence, suggesting that the transcripts derived from this region could adopt different RNA secondary structures. LINE-1 insertions within genes are biased toward the antisense orientation (Smit 1999; Flasch et al. 2019; Sultana et al. 2019), which suggests that for an important number of LINE-1 copies, full-length antisense LINE-1 RNA synthesis could be driven by the host gene promoter.

SINEs make up ∼13% and ∼8% of the human and mouse genome, respectively. Several families (e.g., human Alu and SVA elements, and mouse B1 and B2 elements) are still active and hijack the LINE-1-encoded proteins to be mobilized in *trans* (Dewannieux et al. 2003, 2005; Hancks et al. 2011). The most abundant SINEs elements, human Alu and mouse B1 and B2, are respectively ∼300, ∼135, and ∼200 bp-long, and are all transcribed by RNA-polymerase III (for review, see Richardson et al. 2015). However, Alu elements are highly prevalent in RNA-polymerase II transcripts, due to their preference for inserting in gene-rich regions (Lander et al. 2001). Within genes, Alus are generally located in introns and UTRs, in both sense and antisense orientations, suggesting that two different Alus present in the same transcript could form intramolecular dsRNA interactions (Deininger 2011).

DsRNA structures adopted by TEs resemble viral RNAs, which cells can aberrantly recognize as invading viruses and trigger the type I interferon (IFN) response. In the next section, the type I IFN response is discussed, and more specifically, the types of nucleic acids and receptors involved in their recognition.

## THE TYPE I INTERFERON RESPONSE AND NUCLEIC ACID SENSING MECHANISMS: AN OVERVIEW

In mammals, the type I IFN response constitutes the major innate defense mechanism against viruses, and it is triggered after sensing the presence of viruses. To this end, mammalian cells have evolved a plethora of sensors that recognize typical traits of viral nucleic acids. Upon detection of viral nucleic acids, signaling pathways that culminate in the expression of type I IFNs and proinflammatory cytokines are activated. Secreted IFNs act in both a paracrine and autocrine manner. By binding the IFN receptor (IFNAR1/2) and stimulating the JAK/STAT pathway, IFNs trigger the expression of hundreds of interferon-stimulated genes (ISGs). ISGs are responsible for establishing an antiviral cellular state to prevent viral infection and replication as well as stimulating the adaptive immune response to generate long-lasting antiviral immunity (Schneider et al. 2014; Ivashkiv and Donlin 2015).

In this section, we will describe the nucleic-acid sensing mechanisms present in mammalian cells. Nucleic acid sensors localize in the cytoplasm, the cell surface, and endosomal compartments to detect the presence of viral genomes or replication intermediates. While most cytoplasmic sensors are widely expressed, the expression of endosomal receptors is generally restricted to specific cell types. Besides initiating the type I IFN response, nucleic acids sensors can also activate other antiviral pathways, such as the host translational shutoff. Here, we will summarize the different mammalian nucleic acid-sensing pathways based on the types of virus-derived nucleic acids they recognize, and that are relevant for the focus of this review.

### Double- and single-stranded RNA

**FIGURE 2.**
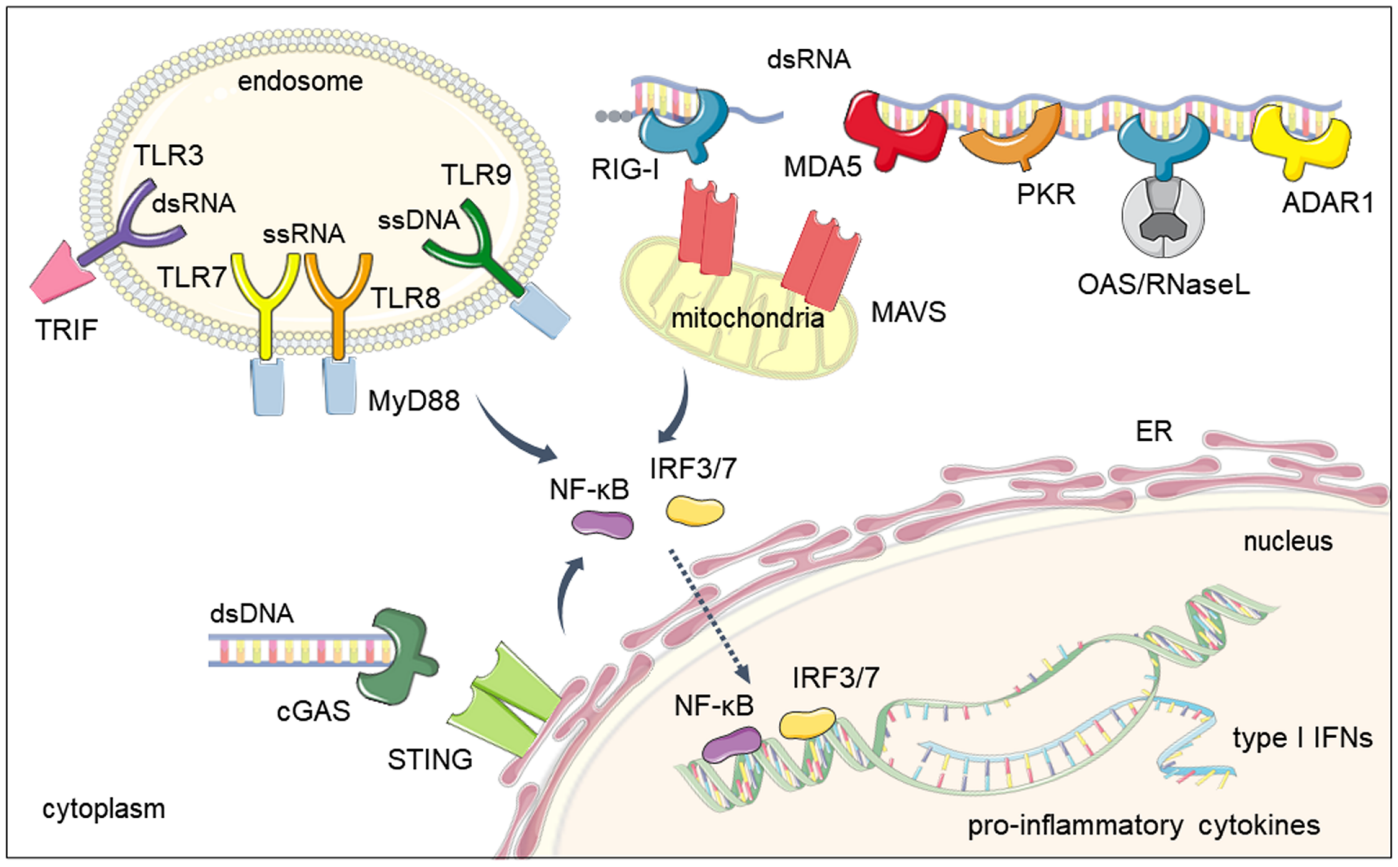
Mammalian nucleic acids sensors. Sensors are classified depending on their subcellular localization and the type of nucleic acid they recognize (DNA vs. RNA). Toll-like receptors (TLRs) usually reside at the cell membrane or the endosome and recognize several types of nucleic acids: TLR3 for dsRNA, TLR7-8 for ssRNA, and TLR9 for ssDNA and RNA/DNA hybrids. cGAS and the RIG-I-like (RLR) family of receptors are typically cytoplasmic, although cGAS can also be found in the nucleus. cGAS recognizes DNA, while RLRs, MDA5 and RIG-I recognize long or short dsRNA molecules, respectively. Upon nucleic acid recognition, these receptors trigger a signalling cascade that promotes nuclear translocation of the IRF3/7 and NF-kB transcription factors and expression of type I IFNs and proinflammatory cytokines. Other cytoplasmic proteins recognize virus-derived dsRNA to induce the host translational shutoff response (PKR), widespread cytoplasmic RNA degradation (OAS/RNase L) and deamination of dsRNA molecules (ADAR1).

The presence of cytoplasmic dsRNA is the hallmark of viral infections and active viral replication (Kumar and Carmichael 1998). Virus-derived dsRNAs are sensed by the cytoplasmic retinoic acid-inducible gene I (RIG-I)-like receptors (RLR), which are composed of the three ATP-dependent DExD/H box RNA helicases, RIG-I, MDA5 (melanoma differentiation-associated gene 5), and LGP2 (laboratory of genetics and physiology 2). RIG-I preferably binds short stretches of dsRNA (<300 bp) bearing 5′-di/triphosphate groups (Hornung et al. 2006; Pichlmair et al. 2006; Myong et al. 2009; Schmidt et al. 2009; Goubau et al. 2014), while MDA5 binds long molecules of dsRNA (>1 Kbp) (Kato et al. 2008; Feng et al. 2012). Both RIG-I and MDA5 contain amino-terminal caspase activation recruitment domains (CARD) that interact with the mitochondrial antiviral signaling (MAVS) protein to activate the nuclear translocation of the transcription factors interferon regulatory factor 3 (IRF3) and IRF7 and nuclear factor kB (NF-kB) to trigger the expression of type I IFNs and proinflammatory cytokines such as interleukin 6 (IL-6), IL-8 and tumor necrosis factor α (TNF-α) (Fig. 2; Kawai et al. 2005; Seth et al. 2005). Despite the ability of LGP2 to bind dsRNA, the absence of CARD domains prevents the signal transduction for IFN production and it is assumed that the main role of LGP2 is regulating the signaling activity of both RIG-I and MDA5 (Yoneyama et al. 2005; Deddouche et al. 2014).

Type I IFN expression can also be triggered by toll-like receptor 3 (TLR3)-mediated dsRNA recognition. This sensor belongs to the toll-like family receptor (TLR) and, depending on the cell-type, is localized at the cell membrane or endosomal compartment. TLR3 binding to dsRNA activates a TRIF-dependent signaling pathway that culminates in expression of type I IFNs and proinflammatory cytokines (Alexopoulou et al. 2001; Oshiumi et al. 2003). Other members of the TLR family recognize viral RNA of a single-stranded conformation (ssRNA). Human TLR7 senses ssRNA and TLR8 binds ssRNA in conjunction with products of RNase degradation to trigger type I IFN expression in a myeloid differentiation primary response 88 (MyD88)-dependent pathway (Fig. 2; Diebold et al. 2004; Heil et al. 2004; Greulich et al. 2019; Ostendorf et al. 2020).

Besides IFN production, the presence of dsRNA in the cytoplasm of cells can activate other pathways that are important for antiviral defense. These include the activation of the inflammasome, the host translational shutoff, the formation of stress granules and widespread RNA degradation. Recognition of dsRNA by the DEAH-box helicase 33 (DHX33) activates the NLR family pyrin domain containing 3 (NLRP3) inflammasome, a proinflammatory pathway characterized by the production of IL-1β and IL-18 (Mitoma et al. 2013). Upon dsRNA binding, the interferon-inducible protein kinase R (PKR) phosphorylates the initiation factor of translation eIF2α to block cap-dependent mRNA translation of viral and host mRNAs in a nonselective manner (Levin and London 1978). The inhibition of translation also results in the formation of stress granules in response to viral dsRNA accumulation (Kedersha et al. 1999; Okonski and Samuel 2013). These cytoplasmic foci are composed of untranslated mRNAs and translational incompetent complexes (Miller 2011). Finally, the IFN-stimulated oligoadenylate synthetase (OAS)/RNase L system, whose activity is also triggered by the presence of virus-derived dsRNA, causes widespread degradation of cytoplasmic RNAs (Kumar and Carmichael 1998). Activation of OAS enzymatic activity by dsRNA results in the generation of 2′–5′ oligoadenylates. These oligomers promote the dimerization and activation of RNase L, an endonuclease that causes widespread degradation of cytoplasmic ssRNA, thus also promoting the translational arrest characteristic of infected cells (Floyd-Smith et al. 1981; Kodym et al. 2009; Burke et al. 2019; Rath et al. 2019). Interestingly, the OAS/RNase L system was also shown to be involved in controlling LINE-1 and IAP retrotransposition levels (Zhang et al. 2014).

Other IFN-stimulated dsRNA binding proteins that are important in regulating type I IFN activation include the adenosine deaminase acting on RNA 1 (ADAR1), which converts adenosines to inosines in dsRNA molecules (George et al. 2014). ADAR1 activity results in the destabilization of dsRNA structures, thus preventing RLR-dependent activation of the type I IFN response (Mannion et al. 2014). This activity has been shown to be important for destabilizing dsRNA-structures formed by Alu elements to avoid their recognition as virus-derived dsRNAs (Chung et al. 2018).

### Double- and single-stranded DNA

The major pathway for cytoplasmic viral DNA sensing is initiated by the cyclic GMP-AMP synthase (cGAS) (Sun et al. 2013). Stimulation of cGAS activity by dsDNA results in the formation of cyclic-GMP-AMP (cGAMP) and signaling through the endoplasmic reticulum-associated stimulator of interferon genes (STING) protein. Analogous to RLR-signaling, the cGAS/STING pathway promotes IRF3 and NF-kB nuclear translocation to induce type I IFN and proinflammatory cytokines expression (Wu et al. 2013). Although activation of cGAS is dependent on the length of dsDNA, this sensor can also bind stem–loop structures formed within ssDNA or DNA/RNA hybrids (Fig. 2; Mankan et al. 2014; Herzner et al. 2015; Luecke et al. 2017). cGAS has been classically considered a cytoplasmic sensor, but it can also localize in the nucleus and recognize viral-derived DNA in this compartment (Lahaye et al. 2018).

The endosomal TLR9 receptor preferentially recognizes unmethylated ssDNA (Hemmi et al. 2000) but is also stimulated by DNA/RNA hybrids (Rigby et al. 2014). Similarly to TLR7 and 8, TLR9 signals in a MyD88-dependent manner leading to the production of type I IFNs and proinflammatory cytokines through the activation of transcription factors IRF3/-7 and NF-kB (Fig. 2; Kawai et al. 2004; Hoshino et al. 2006).

Viral dsDNA can also trigger the absent in melanoma 2 (AIM2)-inflammasome resulting in the production of IL-1β and IL-18, as well as pyroptosis, a caspase-1 dependent cell death program associated with inflammation (Fernandes-Alnemri et al. 2009; Rathinam et al. 2010). In mice, all AIM2-like receptors are dispensable for the type I IFN response to intracellular DNA (Gray et al. 2016).

Besides IFN production and inflammasome activation, the presence of cytoplasmic DNA is sensed by the family of cytidine-deaminases known as apolipoprotein B mRNA-editing enzyme or APOBEC (Salter et al. 2016). Their enzymatic activity catalyzes the conversion of cytidines to uridines in ssDNA, as well as in RNA. This activity has been shown to hypermutate the cDNA of both HIV-1 and retrotransposons (LINEs, SINEs, and ERVs), suggesting that APOBECs have a role in defending against both endogenous and exogenous genomic parasites (Harris et al. 2003; Esnault et al. 2005; Chiu et al. 2006; Muckenfuss et al. 2006; Carmi et al. 2011; Richardson et al. 2014).

The APOBEC, ADAR, and RNase L examples illustrate the substantial overlap between the mechanisms guarding defence against viruses and TEs. In addition, TE-derived nucleic acids can be sensed by cellular nucleic acid sensors and trigger the type I IFN response, pointing out that TE activity needs to be tightly controlled to improve the accuracy of antiviral defence.

## TE-DERIVED NUCLEIC ACIDS AS TRIGGERS OF THE TYPE I IFN RESPONSE

There is mounting evidence of TEs acting as substrates for the classical viral-nucleic acid sensors in fields ranging from autoimmune diseases to cancer. Here we will summarize the literature reporting TE-mediated IFN activation and discuss some of the questions that these findings raise considering the current knowledge in TE biology and nucleic acid sensing.

### Cancer

Several types of cancer show reactivation of ERVs and LINEs expression, a feature that is considered to contribute to cellular transformation (Burns 2017; Bannert et al. 2018). The transcriptional silencing of both types of TEs in healthy cells is controlled by DNA methylation, KRAB-zinc finger proteins and histone repressive marks (Rowe and Trono 2011). Tumors are generally characterized by low DNA CpG methylation levels compared to healthy tissues. Considering that most methylated CpG sites are concentrated in the highly repetitive sequences of the genome, the expression of ERVs and LINEs is, as a consequence, reactivated in cancers (Ehrlich 2009; Burns 2017). Counterintuitively, DNA-methylation inhibitors are used as therapeutic agents in specific types of cancer, such as hematological malignancies, raising the question of what the molecular consequences of further demethylation of cancerous cells are. Two independent reports showed that treatment of colorectal and ovarian carcinoma cells with low doses of the demethylating agent 5-aza-2′-deoxycytidine (5-aza) led to the accumulation of dsRNAs and subsequent activation of the IFN response (Chiappinelli et al. 2015; Roulois et al. 2015). In colorectal cancer, 5-aza treatment resulted in activation of the IFN response in a MDA5/MAVS-dependent manner with a concomitant up-regulation of ERV expression (Roulois et al. 2015). In ovarian carcinoma cells, treatment with 5-aza resulted in activation of IFN and ISG expression through the TLR3/MAVS pathway, an effect that was partially rescued by inhibiting the expression of two specific ERVs. Although the relationship between IFNs and cancer is complex, IFNs are positive players in stimulating antitumor immunity (Budhwani et al. 2018; Borden 2019). For instance, treatment with demethylating agents slowed the population doubling time of colorectal cancer cells in vitro and their ability to develop tumors after injection in nude mice, and importantly, these effects were abrogated by preventing IFN activation. In a mouse model of melanoma, 5-aza sensitized cells to anti-CTLA4 immune checkpoint therapy (Chiappinelli et al. 2015). All these results suggest that dsRNA-mediated immune activation of the RLR pathway could constitute a novel therapeutic target to control tumor growth. Other chemotherapeutic agents, such as doxorubicin and anthracyclines have also been shown to trigger a TLR3-dependent IFN activation (Sistigu et al. 2014). The chemotherapeutic agent RRx-001 was also reported to induce an IFN and ISG response in a human colon carcinoma cell line. Treatment with RRx-001 caused accumulation of dsRNA, as revealed by anti-dsRNA immunofluorescence (J2 antibody), and a concomitant reactivation of the expression of endogenous retroviral genes (Zhao et al. 2017). Similarly, loss of proteins responsible for ERV silencing, such as SETDB1, Trim24, and KAP1, resulted in IFN activation in cancerous and noncancerous settings upon accumulation of ERV and LINE transcripts (Herquel et al. 2013; Cuellar et al. 2017; Tie et al. 2018).

Only recently, failure to epigenetically silence LINE-1 retrotransposon expression has been associated with IFN activation. The absence of p53, the most commonly mutated tumor suppressor gene in cancer, resulted in increased expression of locus-specific copies of LINE-1 and was accompanied by an inflammatory signature. This activation of the immune response was reverted by treatment with the reverse transcriptase inhibitor (RTi) lamivudine (3TC) (Tiwari et al. 2020). RTis are compounds that can disrupt the replication cycle of both exogenous retroviruses and endogenous retrotransposons by interfering with the reverse transcription step. Some RTis, such as lamivudine and emtricitabine, are selective and only inhibit LINE-1 RT (Banuelos-Sanchez et al. 2019). These findings suggest that the nucleic acid responsible for IFN activation in the absence of p53 is the product of LINE-1 retrotranscription (Tiwari et al. 2020). Previously, p53-deficient mouse embryonic fibroblasts were found to accumulate mitochondrial-derived dsRNAs capable of triggering IFN expression (Wiatrek et al. 2019), and SINE B1 and B2 elements, which led to a “suicidal” IFN response (Leonova et al. 2013). These findings suggest that several types of TEs and other endogenous nucleic acids could be responsible for triggering the IFN response in the context of p53 deficiency. An alternative epigenetic pathway controlling LINE-1 expression is orchestrated by the human silencing hub (HUSH) complex. The expression of the central subunit, MPP8, was found to be decreased in cancer and correlated with tumors displaying an immune signature. Loss of the HUSH complex resulted in increased LINE-1 expression and IFN activation in a RLR/MAVS-dependent manner, suggesting that LINE-1 dsRNA can also act as a trigger of the IFN response (Tunbak et al. 2020).

**FIGURE 3.**
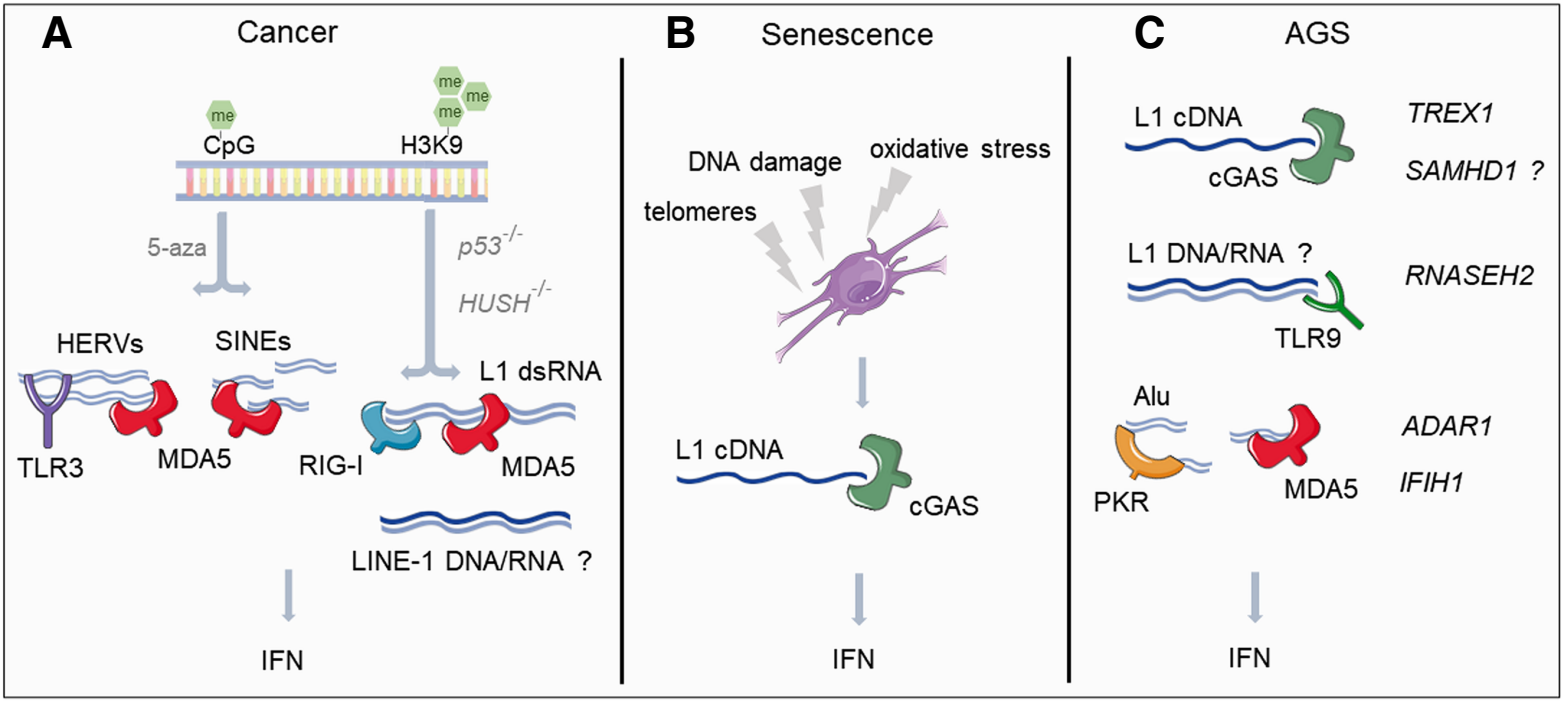
Recognition of TEs as viral nucleic acids in cancer, senescence, and Aicardi–Goutières syndrome (AGS). (*A*) Treatment of cancer cells with demethylating agents (5-aza) or genetic manipulation of factors involved in epigenetic repression (HUSH complex) or genetic stability (p53) leads to increased expression of different types of retrotransposons, including HERVs, LINEs, and SINEs. The RNA molecules derived from these elements are recognized by nucleic acid sensors, triggering the type I IFN response. (*B*) Changes in the genomic architecture of senescent cells are responsible for increased expression of LINE-1 elements. In senescence, the sensor cGAS recognizes the retrotranscription product (cDNA) of LINE-1 triggering IFN expression. (*C*) Mutations in genes involved in nucleic acid metabolism (*TREX1*, *SAMHD1*, *RNASEH2A/B/C*, and *ADAR1*) and nucleic acid sensing (*IFIH1*, which encodes MDA5) are associated with AGS, a disease characterized by IFNs expression in the absence of infections. LINE-1 cDNA accumulation in the absence of functional TREX1 has been suggested to trigger IFN expression in a cGAS-dependent manner, this mechanism could also operate in the context of *SAMHD1* mutations. The accumulation of unedited dsRNA Alu molecules in the absence of functional ADAR1 triggers IFN expression in a MDA5-dependent manner and the host translational shutoff by PKR. Gain-of-function mutations of *IFIH1* lead to recognition of dsRNA-Alu molecules as nonself and consequent IFN activation. The role of TE in the context of *RNASEH2* mutations is still unclear, but, hypothetically, accumulation of LINE-1 RNA/DNA hybrids could be triggering the IFN response in this context.

It is becoming increasingly evident that the expression of dsRNA-forming loci is activated after treatment with DNA demethylating agents or inactivation of epigenetic repressors (Fig. 3A). However, the exact origin of the nucleic acids responsible for the immune activation is still unclear. A recent attempt to answer this question found that the innate immune sensor MDA5 recognizes Alu elements from colorectal cancer cells in vitro, despite Alu expression not being increased after low-dosage 5-aza treatment (Mehdipour et al. 2020). Therefore, a more targeted search for in vivo RNA ligands of the RLR receptors, for instance using CLIP-seq (cross-linking and immunoprecipitation coupled to high-throughput sequencing) or similar techniques, will help to elucidate the identity of the specific substrates for activating the IFN response in cancer after demethylating treatment.

### Cellular senescence

Cellular senescence is an irreversible state of cell cycle arrest associated with aging, but also wound healing and development, typically triggered in response to stress or damaging agents. For instance, telomere shortening, mitochondrial dysfunction, oxidative stress and DNA damage can trigger senescence (Hernandez-Segura et al. 2018). Apart from the permanent cell cycle arrest, the senescent state is characterized by the “senescence-associated secretory phenotype” (SASP), where cells release cytokines, chemokines, extracellular matrix components, and growth factors (Novakova et al. 2010; Lopes-Paciencia et al. 2019). Senescence is also accompanied by changes in the genome architecture, resulting in the loss of repressive epigenetic marks and heterochromatin formation and consequent increased expression of the human retrotransposons Alu, SVA, and LINE-1 (Wang et al. 2011; De Cecco et al. 2013; Van Meter et al. 2014). The increase in retrotransposon expression has been observed in different models of in vitro-induced cellular senescence. High cell-passage numbers, oxidative stress, hypomethylation (5-aza) and treatment with chemotherapy agents (adriamycin) resulted in dysregulation of TE expression, with LINE-1 and ERV-1 elements being the predominant up-regulated biotypes. Interestingly, TE up-regulation was found to be accompanied by activation of the proinflammatory phenotype (SASP) and the type I IFN response (Colombo et al. 2018). Using a model of late senescence, LINE-1 was found to be responsible for activation of the type I IFN response in a cGAS/STING-dependent manner (Fig. 3B). Treatment of cells with shRNAs against LINE-1 or the RTi lamivudine reverted the activation of the IFN response, suggesting that the LINE-1 cDNA was responsible for triggering IFN expression (De Cecco et al. 2019). Similar results were reported in a mouse model of premature aging. SIRT6 knockout mice display increased genomic instability and a premature aging phenotype which is accompanied by increased LINE-1 expression and a cGAS-dependent activation of the type I IFN response (Mostoslavsky et al. 2006; Van Meter et al. 2014; Simon et al. 2019). Many of the phenotypes associated with SIRT6 deficiency, such as premature aging, colitis and increased IFN expression, were reverted after treatment with RTis, suggesting a central role for the LINE-1/IFN interaction in the disease-associated traits of SIRT6 KO mice (Simon et al. 2019).

An additional exciting observation is the requirement of an active IFN response for establishing a full senescent phenotype. Blocking IFN signaling by knocking out the IFN receptor (IFNAR) abolished the establishment of a fully mature SASP response (De Cecco et al. 2019). Interestingly, the cGAS/STING pathway has also been shown to be necessary to establish senescence (Glück et al. 2017; Yang et al. 2017). The exact role of the IFN response in promoting a full-senescent phenotype remains an open question.

One common observation from these reports is the proposed role for cytoplasmic-localised LINE-1 cDNA as the molecule responsible for triggering IFN expression in a cGAS-dependent manner. Considering that the accepted model for the LINE-1 lifecycle suggests that the retrotranscription step occurs in the nucleus, further experimentation is necessary to reconcile these findings. It is possible that senescent cells localize both LINE-1 retrotranscription and/or cGAS in different subcellular compartments than actively cycling cells. For instance, senescent cells could be retrotranscribing LINE-1 in the cytoplasm, if cytoplasmic RNA/DNA molecules could act as primer. Only recently, Alu elements have been shown to be retrotranscribed in the cytoplasm by the LINE-1-encoded machinery independently of its retrotransposition, bringing closer the possibility that LINE-1 RNA could be also used as template to generate LINE-1 cDNA in the cytoplasm (Fukuda et al. 2021). Alternatively, LINE-1 cDNA could also be recognized by cGAS in the nucleus, as a significant proportion of this sensor has been found in the nuclear compartment of specific cell types (Xia et al. 2018; Gentili et al. 2019; Volkman et al. 2019). It is important to note that nuclear cGAS does not react to genomic DNA because its binding to nucleosomes maintains this sensor in an “inactive” state and incompetent for signaling (Zierhut et al. 2019; Boyer et al. 2020; Cao et al. 2020; Kujirai et al. 2020; Pathare et al. 2020). In addition, BAF, a chromatin-associated protein, has also been recently shown to compete cGAS for dsDNA binding, thus preventing recognition of self-DNA (Guey et al. 2020). Considering that newly retrotranscribed LINE-1 cDNA will lack chromatinization during de novo insertion, we hypothesize that this could provide a short window of opportunity for nuclear cGAS to sense the LINE-1 cDNA as an invading pathogen. It will be interesting to measure BAF levels in senescent cells as well as the nuclear envelope integrity, but also test whether nuclear/cytoplasmic cGAS is responsible for recognizing the LINE-1 cDNA or DNA/RNA intermediate during the reverse transcription step. This will help to clarify how LINE-1 cDNA can trigger the cGAS–STING sensing pathway.

### Autoimmune diseases

In recent years, TEs-derived nucleic acids have attracted much attention as molecules that may be responsible for driving inflammation in the context of autoimmune diseases. In this section, we will summarize the literature reporting TE-mediated immune activation in disease, both in the context of the rare autosomal recessive autoinflammatory disorder Aicardi–Goutières syndrome and the much more common autoimmune disease, systemic lupus erythematosus.

#### Aicardi–Goutières syndrome

Aicardi–Goutières syndrome (AGS) is a heritable form of inflammatory encephalopathy with symptoms resembling congenital viral infections of the brain (Crow et al. 2014), including a characteristic elevation of type I IFN levels both in serum and cerebrospinal fluid of patients (Goutières et al. 1998). Mutations in different genes involved in the metabolism or sensing of nucleic acids can lead to AGS (Crow and Manel 2015; Rice et al. 2020). Therefore, AGS has become the paradigm in the study of the role of aberrant accumulation/sensing of endogenous nucleic acids as triggers of the IFN response. Although the precise origin of the nucleic acids driving IFN activation is still unclear, here, we will summarize the current evidence supporting the hypothesis that retrotransposition-intermediates (RNA and DNA) can act as the immunostimulatory molecules triggering the type I IFN response in AGS.

Gain-of-function mutations in the sensor *IFIH1* and partial loss of function mutations in *TREX1, SAMHD1*, and *ADAR1* genes, as well as the genes encoding for the three subunits of the RNase H2 complex can lead to AGS. Besides the classical function of most of these genes in regulating different aspects of nucleic acids metabolism, *TREX1, SAMHD1*, and *ADAR1* have also been implicated in restricting the mobilization of LINE-1 retrotransposons (Stetson et al. 2008; Zhao et al. 2013; Orecchini et al. 2016; Li et al. 2017a). Intriguingly, RNase H2 has been described both as a promoter as well as an inhibitor of LINE-1 retrotransposition (Bartsch et al. 2017; Benitez-Guijarro et al. 2018). The role of these genes in controlling retrotransposition suggests that their loss of function in AGS may lead to increased accumulation of TE-intermediates that could trigger innate immune activation in an RNA (ADAR1) or DNA-dependent (TREX1, RNase H2, and SAMHD1) manner (Fig. 3C). Below, we summarize the proposed roles for proteins mutated in AGS on the control of TEs.

##### TREX1

This gene encodes a 3′ to 5′ DNA exonuclease whose expression is controlled by IFNs (ISG) (Mazur and Perrino 2001; Grieves et al. 2015). *TREX1* loss of function or dominant negative mutations have been reported in AGS, familial chilblain lupus and systemic lupus erythematosus patients (Crow et al. 2006a; Lee-Kirsch et al. 2007; Rodero and Crow 2016). One of the first animal models for *Trex1* deficiency revealed that this enzyme is required to prevent a lethal inflammatory phenotype in mice (Morita et al. 2004). The cGAS/STING-dependent autoinflammatory phenotype of *Trex1*-deficient mice correlated with the accumulation of ssDNA from LINE-1, LTR endogenous retroviruses and SINE elements (Stetson et al. 2008; Gall et al. 2012; Gray et al. 2015). Supporting the notion that Trex1 may regulate the levels of retrotransposition byproducts, in vitro retrotransposition assays showed reduced mobilization of human LINE-1 element and the mouse IAP LTR retrotransposon during Trex1 overexpression (Stetson et al. 2008). All this led to the hypothesis that mouse Trex1 metabolizes the ssDNA products of TE retrotranscription, preventing their accumulation and consequent activation of the innate immune response.

In humans, neurons, neuronal progenitor cells (NPCs) and astrocytes derived from *TREX1*-deficient human stem cells also displayed an increase in intracellular DNA species, including LINE-1 elements and elevated type I IFN levels. The accumulation of LINE-1 cDNA and consequent IFN activation was proposed to be responsible for the neurotoxic phenotype, as this was reverted after treatment with the RTis or after antagonizing IFN signaling (Thomas et al. 2017). These results suggest that in both humans and mice, the absence of TREX1 can lead to LINE-1 cDNA accumulation triggering the IFN response through the cGAS–STING pathway (Fig. 3C). However, this hypothesis has been challenged by the observation that treatment of *Trex1*^−/−^ mice with RTis did not reduce the spontaneous ISG signature, suggesting that retrotransposon-independent mechanisms may also contribute to the inflammatory phenotype (Achleitner et al. 2017).

##### RNase H2

The RNase H2 complex is composed of the three different subunits: RNASEH2A, RNASEH2B, and RNASEH2C. RNase H2 activity is essential for genome stability and mammalian embryonic development (Hiller et al. 2012; Reijns et al. 2012). This ribonuclease initiates the removal of ribonucleotides incorporated in genomic DNA during replication, and it is also thought to be involved in resolving R-loops formed during transcription, by catalyzing the degradation of RNA in RNA/DNA hybrids. Biallelic mutations in the genes encoding the three subunits are the most common cause of AGS, and monoallelic mutations have been associated with increased risk of systemic lupus erythematosus (Crow et al. 2006b, 2015; Günther et al. 2015). Analysis of mice with partial-loss-of-function AGS disease mutations in *Rnaseh2a* and *Rnaseh2b*, as well as experiments using *Rnaseh2b* knockout cells established that RNase H2 function is necessary to prevent activation of the IFN response and ISG expression in a cGAS/STING-dependent manner (Mackenzie et al. 2016; Pokatayev et al. 2016). For instance, mutation in the highly conserved glycine residue (G37S), near the catalytic center of the RNASEH2A subunit, causes a severe early onset presentation of AGS. In mice, this mutation was shown to activate ISGs expression, with a concomitant accumulation of cytoplasmic LINE-1 DNA. This led to the hypothesis that accumulation of LINE-1 DNA in the absence of functional RNase H2 could lead to activation of the IFN response (Pokatayev et al. 2016). Similarly, a mouse model for the most common missense mutation found in AGS patients (RNASEH2B-A177T), led to ISG activation in a cGAS/STING-dependent manner (Mackenzie et al. 2016). However, the role of RNase H2 in the control of retrotransposons is still controversial, as it has been suggested to be necessary for both restricting and facilitating LINE-1 activity. Restriction of LINE-1 retrotransposition was suggested to involve a physical interaction with the well-known LINE-1 restricting factor MOV10 (Choi et al. 2018), while two other reports contradicted these findings and showed that RNase H2 function is necessary for LINE-1 mobilization (Bartsch et al. 2017; Benitez-Guijarro et al. 2018). The proposed model is that LINE-1 elements rely on the endogenous RNase H activity of cellular RNase H2 to degrade the RNA moiety after reverse transcription and successfully synthesize their second strand cDNA to complete their retrotransposition cycle (Benitez-Guijarro et al. 2018). Considering this model, the absence of functional RNase H2 could lead to the accumulation of unprocessed LINE-1 RNA/DNA hybrids and potentially trigger the IFN response in a cGAS/STING-dependent manner (Fig. 3C; Mankan et al. 2014; Rigby et al. 2014).

Importantly, the absence of RNase H2 also causes a significant increase in DNA damage, widespread DNA hypomethylation, and micronuclei formation, which can trigger the IFN response independent of retrotransposon intermediates (Reijns et al. 2012; Lim et al. 2015; MacKenzie et al. 2017). Therefore, further investigation is necessary to clarify the specific nature of the nucleic acids that trigger IFN expression in the context of RNase H2 deficiency.

##### SAMHD1

Mutations in this gene are found in AGS, as well as in patients with solid tumors and leukemia (Rice et al. 2009; Clifford et al. 2014; Rentoft et al. 2016). This gene encodes a deoxynucleoside triphosphate triphosphohydrolase (dNTPase) that controls dNTP levels in cells and whose expression is controlled by IFNs (ISG). Ensuring optimal dNTPs levels is required for efficient cellular DNA replication and cDNA synthesis of retrovirus and retrotransposons. In the absence of functional SAMHD1, ssDNA fragments from stalled replication forks are released to the cytoplasm where they are thought to activate IFN expression in a cGAS/STING-dependent manner (Coquel et al. 2018). Considering the impact of SAMHD1 on dNTP levels, this enzyme also acts as a restriction factor against the HIV-1 retrovirus and the LINE-1 retrotransposon (Fig. 3C; Lahouassa et al. 2012; Zhao et al. 2013). SAMHD1-mediated restriction of HIV-1 can be ameliorated by dNTP supplementation, but the exact role of SAMHD1 enzymatic activity in the context of LINE-1s remains unclear, as its role in restricting LINE-1 mobilization has been shown to be both dependent and independent of its enzymatic activity (Zhao et al. 2013; Herrmann et al. 2018). For instance, AGS-associated SAMHD1 mutations outside the catalytical sites also fail to restrict LINE-1 mobilization (Zhao et al. 2013). The current proposed model is that SAMHD1 restricts LINE-1 mobilization by both reducing ORF2p levels and LINE-1 reverse transcription, as a consequence. Alternatively, SAMHD1 has been shown to directly interact with ORF2p and locally deplete dNTP levels to prevent LINE-1 retrotranscription at the site of integration (Zhao et al. 2013; Herrmann et al. 2018). In addition to these models, SAMHD1 has also been proposed to inhibit LINE-1 retrotransposition by promoting the sequestration of the LINE-1 RNP in cytoplasmic stress granules (Hu et al. 2015). The exact role of increased retrotransposon intermediates in activating the IFN response in the absence of functional SAMHD1 remains unknown.

##### ADAR1

This gene encodes two different isoforms of the RNA-editing enzyme ADAR1, p110, and p150, and partial loss-of-function mutations have been shown to cause AGS (Rice et al. 2012). While ADAR1 p110 is constitutively expressed and mostly nuclear, the p150 isoform is IFN-inducible and both nuclear and cytoplasmic (Mannion et al. 2015). Both ADAR1 isoforms catalyze the hydrolytic deamination of adenosine to inosine in dsRNA molecules. Editing can result in amino acid recoding when in protein-coding sequences or disrupt important base-pairing interactions in dsRNA secondary structures. High-throughput studies of the human transcriptome demonstrated that the vast majority of edited residues correspond to noncoding regions, with around 90% residing in the primate-specific Alu element (Athanasiadis et al. 2004; Levanon et al. 2004). The high abundance of sense and antisense Alu elements suggest that they are prone to forming dsRNA molecules, which can be deaminated by ADARs. Loss of *Adar1* in mice leads to early embryonic lethality (E11.5–12.5), a phenotype that can be rescued to birth by preventing IFN activation of the RLR-sensing pathway by interfering with MDA5 or MAVS expression (Wang et al. 2000; Hartner et al. 2009; Mannion et al. 2014; Liddicoat et al. 2015; Pestal et al. 2015). These results suggest that, in the absence of ADAR1, unedited dsRNAs accumulate and act as a substrate for the MDA5/MAVS signaling pathway for IFN production (Fig. 3C). Supporting these observations, the accumulation of endogenous immunogenic dsRNAs in the absence of ADAR1 also stimulated the translational shutoff by stimulating PKR activity (Chung et al. 2018) and the OAS/RNase L system (Li et al. 2017b).

ADAR1 has also been shown to control LINE-1 retrotransposition independent of its deaminating activity by binding to the LINE-1 RNP complex and restricting its retrotransposition through direct interaction with the LINE-1 RNA (Orecchini et al. 2016). In other settings, the accumulation of LINE-1 RNA in the absence of ADAR1 was also shown to trigger the IFN response in an RLR-dependent manner (Zhao et al. 2018). These results suggest that both LINE-1 RNA and unedited Alu elements could act as immunostimulatory molecules in the absence of functional ADAR1.

##### IFIH1

This interferon-stimulated gene is the only known factor mutated in AGS that is not directly involved in the metabolism of nucleic acids, as it encodes the dsRNA sensor MDA5. *IFIH1* mutations are also associated with systemic lupus and type I diabetes (Smyth et al. 2006; Gateva et al. 2009; Oda et al. 2014; Rice et al. 2014). Gain-of-function mutations in *IFIH1* have been reported to lead to IFN activation by preventing discrimination of self versus nonself RNA. DsRNAs derived from sense and antisense Alu elements have been proposed to be the preferred endogenous substrate for gain-of-function mutations of MDA5 (Fig. 3C; Ahmad et al. 2018). Mutated MDA5 was shown to bind and oligomerize on inverted Alu repeats, with a preference for inverted repeats that are in close genomic proximity (< 1Kb) and derived from a single transcript, while wild-type MDA5 could only oligomerize on unedited or perfectly paired Alu-dsRNA structures, suggesting that ADAR1-mediated editing of Alu-dsRNAs could act as a protective mechanism to avoid their erroneous recognition as nonself molecules (Ahmad et al. 2018; Mehdipour et al. 2020).

##### Retrotransposon-independent IFN activation in AGS

Despite the increasing evidence of retrotransposon intermediates as drivers of the IFN response in AGS, an alternative model exists which suggests that IFN expression is activated by DNA damage. Constitutive DNA damage signaling is associated with cell cycle delay, cellular senescence, and up-regulation of IFNs and ISGs. Importantly, DNA damage and genomic instability are phenotypes widely reported upon mutation of several AGS-genes, including *TREX1* (Yang et al. 2007), *RNASEH2A*, *RNASEH2B*, *RNASEH2C* (Hiller et al. 2012; Reijns et al. 2012; Uehara et al. 2018), and *SAMHD1* (Günther et al. 2015; Kretschmer et al. 2015). For instance, cells lacking SAMHD1 showed accumulation of DNA damage and ssDNA fragment in the cytosol, where they activated the cGAS/STING pathway to induce expression of type I IFNs (Coquel et al. 2018). Interestingly, RNase H2-deficient cells accumulated cytosolic DNA aggregates virtually indistinguishable from micronuclei (Bartsch et al. 2017), which colocalized with the nucleic acid sensors cGAS inducing a cGAS/STING-dependent innate immune response (Mackenzie et al. 2016, 2017). These findings point toward a link between AGS-related genes and the DNA-damage response through self-DNA becoming exposed in the cytosol and consequent IFN activation.

The hypothesis that unrestricted retrotransposon activity contributes to AGS is supported by the observation that RTis can treat and even prevent disease-associated phenotypes of AGS. However, treatment of *Trex1*-null mice with both specific and nonspecific LINE-1 RTis led to confounding results. Beck-Engeser et al. reported an improved survival and reduced inflammatory infiltrates in the heart after combinatorial RTi treatment (Beck-Engeser et al. 2011), but Achleitner et al. failed to reproduce these results (Achleitner et al. 2017). In the human context, RTis rescued the neurotoxicity phenotype of AGS-associated TREX1 mutations (Thomas et al. 2017). Recently, stavudine (d4T) was shown to effectively block LINE-1 activity and decrease STING activation after hypoxia-ischemia, reducing neurodegeneration (Gamdzyk et al. 2020). Importantly, a clinical trial using a similar strategy (ClinicalTrials.gov identifier: NCT02363452) reported positive effects of a therapy consisting of a combination of three RTis (lamivudine, abacavir, and zidovudine) in patients with AGS. Treatment resulted in a reduction in the IFN score and IFN-α protein levels. Interestingly, this effect was greatest among the four patients with mutations in components of the RNase H2 complex (Rice et al. 2018). These results support the hypothesis that RTi therapy can prevent IFN signaling in AGS patients by inhibiting reverse transcription driven by endogenous retrotransposons. However, RTis have also been reported to provide an intrinsic anti-inflammatory effect independent of TLRs, the cGAS/STING pathway and its reverse transcriptase inhibition activity (Tarallo et al. 2012; Kerur et al. 2013; Fowler et al. 2014). Taken together, the available data suggest that accumulation of reverse-transcribed DNA species results in an inflammatory phenotype mediated by the cGAS/STING pathway which can be reversed by RTis; however, the exact contribution of their intrinsic anti-inflammatory effect remains unclear.

There are other unanswered questions regarding the mechanisms underlying the induction of the IFN response by retrotransposon-intermediates in AGS and senescence. For instance, it is unclear how the LINE-1 cDNA accumulates in the cytoplasm of AGS cellular models to engage with cGAS for IFN activation. As previously suggested, one possibility is that the LINE-1 cDNA is synthesized directly in this cellular compartment. For this, the LINE-1 RNA and encoded proteins might perform reverse transcription in situ using a nonstandard primer in the cytoplasm.

We propose a model in which by-products of active endogenous retrotransposons in addition to the DNA damage associated with some AGS-mutations are drivers of the IFN response. Clearly, further work is needed to determine the importance of retroelement activity and the detection of retroelement-derived nucleic acids by innate immune sensors in AGS.

#### Systemic lupus erythematosus

The first noninfectious human disease to be associated with an increase in type I IFN activity was systemic lupus erythematosus (SLE) (Hooks et al. 1979). SLE is an autoimmune disease characterized by the production of autoantibodies targeting nucleic acids and nuclear-associated proteins. A link between AGS and lupus was highlighted by the discovery of *TREX1*, *SAMHD1*, *RNASEH2A/B/C*, and *IFIH1* mutations in SLE patients (Lee-Kirsch et al. 2007; Cunninghame Graham et al. 2011; Ravenscroft et al. 2011; Günther et al. 2015). Crow and Rehwinkel were the first to report the significant overlap between AGS and lupus and highlighted the interplay between nucleic acid metabolism and autoimmune disease (Crow and Rehwinkel 2009). Shortly after, Crow put forward LINE-1 retrotransposons as potential endogenous stimuli triggering the immune response and promoting autoimmunity (Crow 2010). Indeed, TEs are dysregulated in SLE and LINE-1 mRNA transcripts were found to be increased, inducing type I IFN production in vitro through both TLR-dependent and TLR-independent pathways (Mavragani et al. 2016; Kelly et al. 2018). In addition, the relationship between autoimmunity and TEs also involves the production of autoantibodies. Autoantibodies against the RNA binding protein Ro60 are present in individuals with SLE and other autoimmune disorders. Alu elements were found associated with the Ro60 immune complexes from the blood of individuals with lupus (Hung et al. 2015), and autoantibodies against LINE-1 ORF1 are characteristic of a population of SLE patients with severe and active disease and higher type I IFN score (Carter et al. 2020).

Unlike non-LTRs, the potential role of HERV in autoimmunity has been more widely explored. HERV proteins can be recognized by the host as exogenous and trigger an immune response and antibody production. Patients suffering from autoimmune disorders present autoantibodies against the *gag* and *env* regions of retroviruses that can cross-react with endogenous factors. In SLE for example, patients produce autoantibodies against the p30 *gag* protein from the endogenous retrovirus HRES-1 that can cross-react with the common autoantigen U1-70K (Perl et al. 1995). Similar findings in other autoimmune diseases have led to the formulation of a model in which HERV antigens stimulate immunity due to their similarity to exogenous viral proteins, a process termed “molecular mimicry.” Several reviews have extensively discussed the evidence for HERV in the aetiopathogenesis of autoimmune disorders, including lupus, diabetes mellitus, multiple sclerosis, Sjögren's syndrome, and rheumatoid arthritis, among others (Balada et al. 2009, 2010; Brodziak et al. 2012; Morandi et al. 2017; Grandi and Tramontano 2018a,b; Greenig 2019; Morris et al. 2019; Talotta et al. 2020).

## CONCLUDING REMARKS: IS THE RELATIONSHIP BETWEEN TEs AND IFNs RELEVANT FOR EARLY DEVELOPMENT?

The expression of ERVs and non-LTR retrotransposons is generally silenced in mammalian adult tissues; however, expression of retrotransposons is characteristic of germ cells, early embryos and embryonic stem cells (ESCs) (Peaston et al. 2004; Garcia-Perez et al. 2007; Macia et al. 2011; Schumann et al. 2019). The epigenetic reprogramming of cells during early development to erase germline-specific marks and acquire totipotent and pluripotent capacity involves a general demethylated state resulting in increased TE expression. After fertilization, the zygote undergoes several divisions despite being transcriptionally inactive. The embryonic genome becomes active during the two-cell stage in mouse, and eight-cell stage in humans. During these stages, cells are defined as totipotent, as they have the ability to generate a full organism, including the embryonic and extraembryonic tissues. Later in development, during the blastocyst stage (day 3.5 post-fertilization in mice and day 5 in humans), embryonic stem cells, which can be derived from the inner cell mass, are only pluripotent, which means that they can give rise to the three germ layers (ectoderm, endoderm, and mesoderm) that will form the embryo, but not to the extra-embryonic tissue, such as the placenta. Although many mechanisms have evolved to prevent the deleterious effects of TE activity during these stages, the expression of TEs may also be beneficial during early development. Interestingly, the waves of expression pattern of the different classes of transposable elements differs, suggesting that they could contribute to modulating the cellular fate during early development (for reviews, see Gerdes et al. 2016; Torres-Padilla 2020). For example, mouse MERVL is highly transcribed during the two-cell (2C)-stage, and sequences derived from this TE function as promoters to drive the expression of 2C-stage specific transcripts (Macfarlan et al. 2012). The HERVK is expressed during the human embryonic genome activation stage (eight-cell) and sustained during the blastocyst stage. During these stages, the HERVK-encoded protein Rec can bind the 3′UTR of cellular mRNAs promoting their association with polyribosomes (Grow et al. 2015). The expression of another primate-specific transposon, HERVH, is only activated later in development, during the blastocyst stage (Göke et al. 2015). Interestingly, the lncRNA derived from HERVH acts as a scaffold to maintain the pluripotency transcriptional network typical of stem cells through physical interaction with OCT4 and coactivators (Lu et al. 2014). More recently, the RNA from LINE-1 has been suggested to be required for the maintenance of embryonic stem cell self-renewal by silencing the 2C-stage transcriptional program (Percharde et al. 2018).

Despite the high expression of TEs during early development, TE-nucleic acids cannot trigger the IFN response since this pathway is known to be inactive during this stage. ESCs, both from human and mouse origin, are unable to synthesize IFNs upon infections with viruses or when challenged with viral DNA or RNA mimics, and the ability to produce IFNs only becomes active after differentiation (Burke et al. 1978; Chen et al. 2010; Wang et al. 2013; Witteveldt et al. 2019). In agreement, in vivo studies of mouse development confirmed that the ability to produce IFNs only becomes active after early postimplantation (Barlow et al. 1984). The inability of pluripotent cells to produce IFNs has been attributed to both the low expression of the RIG-I-like receptors and the action of miRNAs silencing on MAVS, a central factor for RNA immunity (Wang et al. 2013; Witteveldt et al. 2019). ESCs also fail to produce IFNs in response to cytoplasmic DNA, suggesting that the cGAS/STING pathway is also inactive during pluripotency (Witteveldt et al. 2019). Why the pluripotent stage of development needs to silence the IFN response is unknown, but we hypothesize it may be a requirement to prevent the aberrant production of IFNs upon recognition of TE-derived nucleic acids. In addition, this may be required to avoid the deleterious effects that IFN production has on ESC biology. For instance, constitutive activation of the type I IFN transcription factor IRF7 in ESCs has been shown to drive the expression of a subset of ISGs as well as dysregulation of pluripotency and lineage-specific genes (Eggenberger et al. 2019). All these findings together lead us to hypothesize that TEs have exerted a strong evolutionary pressure to supress the main antiviral response during early mammalian development.
